# Clear cell variant of ameloblastic carcinoma

**DOI:** 10.34172/aim.2022.119

**Published:** 2022-11-01

**Authors:** Varsha V. Kumar, Mamata Kamat, Vasanth Kattimani, Girish H. Channabasaviah, Somashekhar V. Ulasandra

**Affiliations:** ^1^Department of Oral and Maxillofacial Pathology, RajaRajeswari Dental College and Hospital, Bengaluru, Karnataka, India; ^2^Department of Oral Pathology and Microbiology, Bharati Vidyapeeth (Deemed to be University) Dental College and Hospital, Sangli, India; ^3^Department of Oral and Maxillofacial Surgery, District Health and Family Welfare Services, Dharwad, Karnataka; ^4^KVG Dental College, Sullia, Karnataka, India

**Figure 1:**
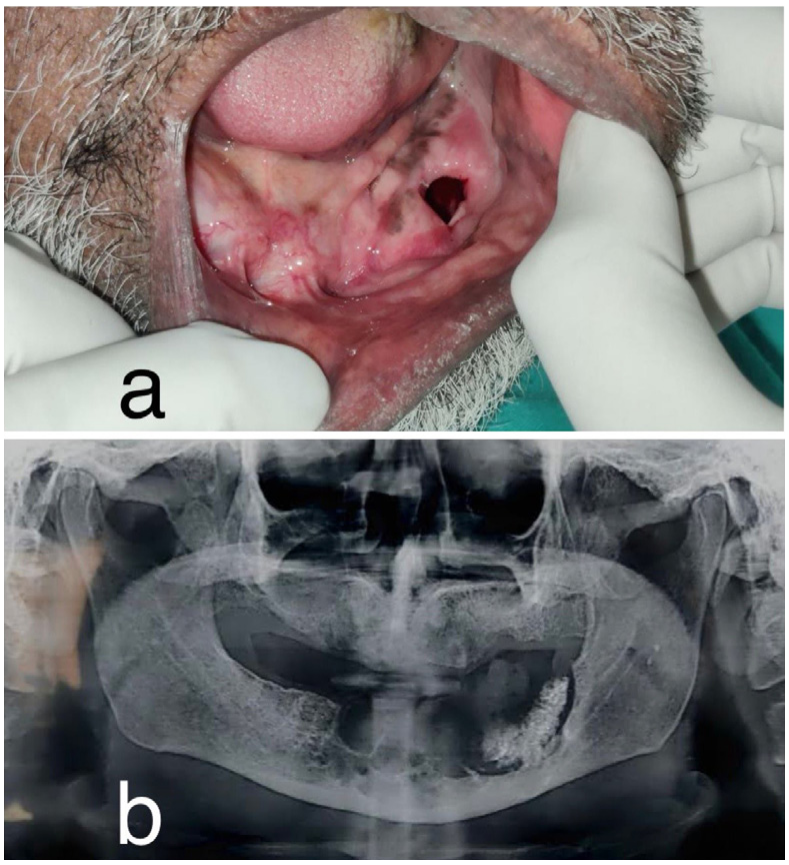
Clinical presentation and radiographic presentation. **(a)** Clinical photograph showing swelling in the edentulous mandible obliterating the lower-left vestibule measuring about 5X4 cm in size and extending to the retromolar region posteriorly. **(b)** Orthopantomogram showing a multilocular radiolucency in the anterior mandible extending posteriorly to the left ramus region.

**Figure 2:**
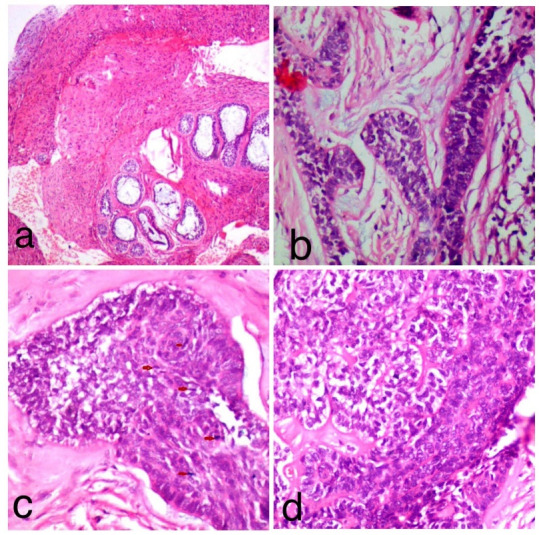
Histopathological examination. **(a) ** Lesion tissue comprised of proliferating odontogenic epithelium in a mature fibro-cellular connective tissue stroma (H& E; 4x). **(b)** High power view showing islands and cords of ameloblastomatous epithelium that showed peripheral tall columnar ameloblast-like cells exhibiting palisading and nuclear polarization, and central stellate-reticulum like cells. Proliferation of malignant odontogenic islands/follicles was seen with budding epithelium. (H & E;10x). **(c)** High power view of malignant odontogenic islands/follicles exhibiting dense cellular arrangement which showed hyperchromatism, nuclear pleomorphism, and mitotic figures as indicated by the red arrow (H & E;40x). **(d)** Focally, odontogenic islands with clear cell differentiation and a zone of hyalinization adjacent to tumor islands are seen. (H & E;40x).

**Figure 3:**
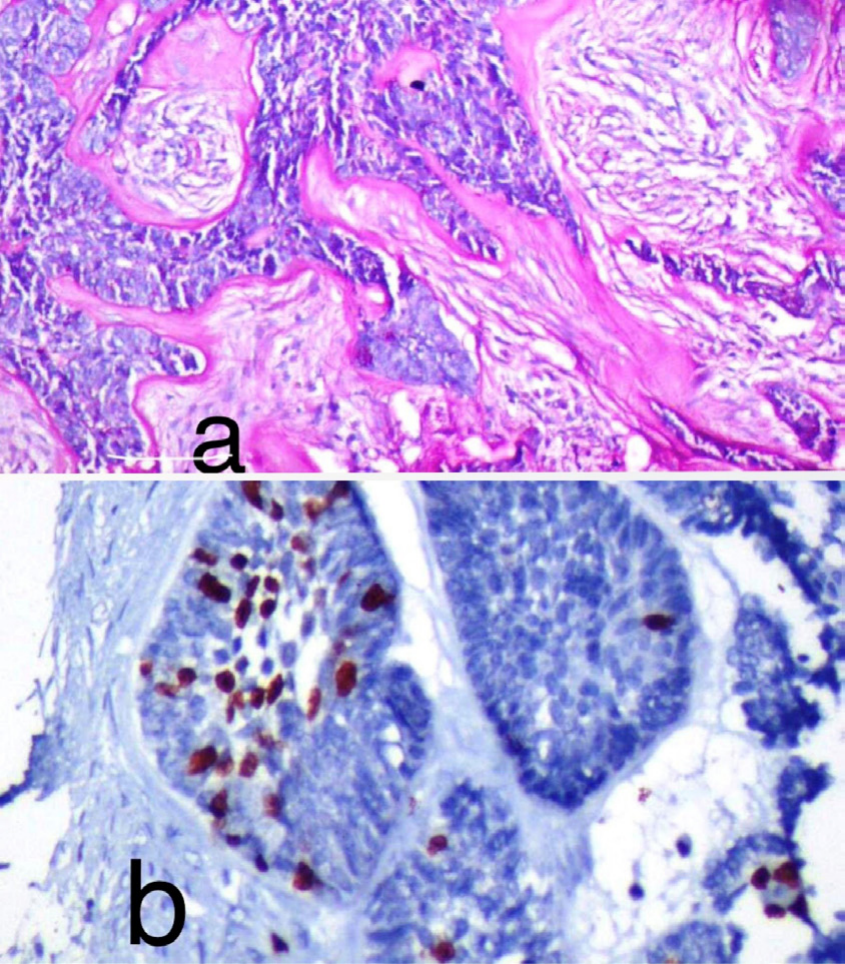
Special staining.** (a)** High power view showing tumor islands with clear cell differentiation positive for PAS. (PAS; 40x). **(b)** Photomicrograph showing Ki-67 immunostaining positivity of tumor cells. (IHC, 10x).

 An 83-year-old male presented with a chief complaint of a gradually increasing swelling over the left lower back region of the jaw of 5 months in duration. The patient mentioned a history of marsupialization of a cystic lesion in the same region 7 years ago. Intra-oral examination revealed an edentulous mandible with a firm, non-tender swelling obliterating the lower-left vestibule measuring about 5 × 4 cm in size and extending to the retromolar region posteriorly (Figure 1a). Orthopantomogram showed a multilocular radiolucency in the anterior mandible extending posteriorly to the left ramus region (Figure 1b).

 Histopathological examination of hematoxylin and eosin-stained sections under scanner view revealed proliferation of odontogenic epithelium in a mature fibro-cellular connective tissue stroma (Figure 2a). Examination under higher magnification revealed islands and cords of ameloblastomatous epithelium that showed peripheral tall columnar ameloblast-like cells exhibiting palisading and nuclear polarization, and central stellate-reticulum like cells. In addition, proliferation of malignant odontogenic islands/follicles was observed with budding epithelium. (Figure 2b). The odontogenic islands showing dense cellular arrangement with hyperchromatism, nuclear pleomorphism and mitotic figures were also evident (Figure 2c). Focally, odontogenic islands with clear cell differentiation and a zone of hyalinization adjacent to tumor islands were seen (Figure 2d). The clear cells stained positive for PAS (Figure 3a). Ki-67 immunohistochemistry showed a proliferative index of 40% (Figure 3b).

 Ameloblastic carcinoma (AC) is a rare, locally aggressive odontogenic malignancy that accounts for < 2% of odontogenic tumors.^1,2^ Two forms of ACs have been recognized: AC arising in pre-existing benign ameloblastoma (carcinoma ex ameloblastoma) and, ACs demonstrating malignant features in the absence of conventional ameloblastoma (*de novo* AC).^3,4^ The most often reported type is carcinoma ex ameloblastoma which is thought to arise when dedifferentiation occurs in ameloblastoma, wherein the aggressive clone becomes dominant, resulting in malignant change.^1,3^ It has been suggested that the surgical manipulation of the lesions like cysts and other benign odontogenic lesions stimulates malignant transformation.^1^ Two-thirds of AC cases occur in the mandible with no gender predilection.^2^ However, some reports have shown male preponderance. Radiographically, it presents as poorly defined multilocular radiolucency with cortical expansion. Microscopically, ACs are characterized by malignant cytologic changes along with areas of conventional ameloblastoma, thus posing a diagnostic challenge.^1,3,5,6^ Hence, adequate sampling of the specimen is of paramount importance to yield the diagnosis of AC. The presence of ameloblastomatous follicles with the budding and anastomosing odontogenic islands that show cellular crowding, hyperchromatism, pleomorphism, mitotic figures, necrosis, etc. will aid in differentiating ACs from benign ameloblastoma.^1^ Additionally, immunohistochemical markers like Ki-67 and p53 are also useful.^1^ In rare instances, ACs show clear cell differentiation within the tumor islands as seen in the present case.^1-4^ These cells are usually PAS-positive confirming the presence of glycogen.^6^ The clear cell variant of AC needs to be differentiated from calcifying epithelial odontogenic tumor (CEOT) with clear cell change, clear cell odontogenic carcinoma (CCOC), salivary gland clear cell adenocarcinoma, and mucoepidermoid carcinoma. CEOT lacks ameloblastomatous and malignant features. CCOC predominantly shows clear cells and lacks ameloblastoma components. This rare clear cell variant of AC has been reported to behave aggressively with high recurrence and poor survival.^1-6^ Hence, definitive diagnosis through adequate sampling, and aggressive treatment along with longer and close follow-up are recommended to prevent recurrence and metastases, and to improve the disease outcome.
